# Selenazolinium Salts as “Small Molecule Catalysts” with High Potency against ESKAPE Bacterial Pathogens

**DOI:** 10.3390/molecules22122174

**Published:** 2017-12-08

**Authors:** Karolina Witek, Muhammad Jawad Nasim, Markus Bischoff, Rosmarie Gaupp, Pavel Arsenyan, Jelena Vasiljeva, Małgorzata Anna Marć, Agnieszka Olejarz, Gniewomir Latacz, Katarzyna Kieć-Kononowicz, Jadwiga Handzlik, Claus Jacob

**Affiliations:** 1 Department of Technology and Biotechnology of Drugs, Faculty of Pharmacy, Jagiellonian University, Medical College, ul. Medyczna 9, 30-688 Cracow, Poland; karolina.witek@uj.edu.pl (K.W.); jawad.nasim@uni-saarland.de (M.J.N.); marcmalgorzata@gmail.com (M.A.M.); agnieszka.olejarz@doctoral.uj.edu.pl (A.O.); glatacz@cm-uj.krakow.pl (G.L.); mfkonono@cyf-kr.edu.pl (K.K.-K.); 2 Bioorganic Chemistry, School of Pharmacy, University of Saarland, Campus B2.1, D-66123 Saarbruecken, Germany; c.jacob@mx.uni-saarland.de; 3 Institute of Medical Microbiology and Hygiene, Saarland University, D-66421 Homburg, Germany; Markus.Bischoff@uniklinikum-saarland.de (M.B.); Rosmarie.Gaupp@uks.eu (R.G.); 4 Department of Medicinal Chemistry, Latvian Institute of Organic Synthesis, Aizkraukles 21, LV-1006 Riga, Latvia; pavel.arsenyan@lycos.com (P.A.); vasiljeva.lena@gmail.com (J.V.)

**Keywords:** selenazolinium salts, ebselen, RSeS, multidrug resistance, MRSA, ESKAPE pathogens, antibacterial agents

## Abstract

In view of the pressing need to identify new antibacterial agents able to combat multidrug-resistant bacteria, we investigated a series of fused selenazolinium derivatives (**1**–**8**) regarding their in vitro antimicrobial activities against 25 ESKAPE-pathogen strains. Ebselen was used as reference compound. Most of the selenocompounds demonstrated an excellent in vitro activity against all *S. aureus* strains, with activities comparable to or even exceeding the one of ebselen. In contrast to ebselen, some selenazolinium derivatives (**1**, **3**, and **7**) even displayed significant actions against all Gram-negative pathogens tested. The 3-bromo-2-(1-hydroxy-1-methylethyl)[1,2]selenazolo[2,3-*a*]pyridinium chloride (**1**) was particularly active (minimum inhibitory concentrations, MICs: 0.31–1.24 µg/mL for MRSA, and 0.31–2.48 µg/mL for Gram-negative bacteria) and devoid of any significant mutagenicity in the Ames assay. Our preliminary mechanistic studies in cell culture indicated that their mode of action is likely to be associated with an alteration of intracellular levels of glutathione and cysteine thiols of different proteins in the bacterial cells, hence supporting the idea that such compounds interact with the intracellular thiolstat. This alteration of pivotal cysteine residues is most likely the result of a direct or catalytic oxidative modification of such residues by the highly reactive selenium species (RSeS) employed.

## 1. Introduction

The emergence and spread of antimicrobial resistance among pathogenic bacteria represents a major global healthcare problem in the 21st century [1]. A number of common pathogens are reported to develop resistances against virtually all types or classes of antibiotics [2]. The most troublesome bacteria that pose a growing challenge for healthcare practitioners due to their antimicrobial resistance are referred as ESKAPE pathogens and include vancomycin-resistant *enterococci* (VRE), methicillin resistant *Staphylococcus aureus* (MRSA), *Klebsiella pneumoniae*, *Acinetobacter baumannii*, *Pseudomonas aeruginosa*, and extended-spectrum β-lactamase (ESBL)-producing or carbapenem-resistant species of the family *Enterobacteriaceae* (CRE). The acronym ESKAPE was first proposed by Rice et al. in 2008 to emphasize the great capacity of these bacteria to “escape” from common antibacterial treatment through rapid acquisition or development of resistance determinants allowing them to tolerate the antimicrobial substance(s) [3,4,5,6]. Each member of the ESKAPE species is a major source of severe and frequently lethal diseases in hospitalized patients, and some of them have also successfully spread to community settings, affecting otherwise healthy individuals [3,7]. Among ESKAPE pathogens, MRSA strains are the most prevalent Gram-positive bacteria, causing nosocomial infections throughout the world [8,9]. Because of their considerable ability to acquire resistance mechanisms against any antibiotics introduced into clinical use, the appearance of MRSA strains in hospitals has been associated with substantial morbidity and mortality rates [9,10,11]. For multidrug-resistant (MDR) Gram-negative bacteria—namely *A. baumannii*, *P. aeruginosa*, *K. pneumoniae*, and *E. coli*, the situation is even more complex and worrisome as “they represent the problem of multidrug resistance to the maximum” [11]. The therapeutic options for infections caused by these MDR pathogens are often so extremely limited that, in many cases, clinicians are left with practically no rational choice of antibiotic treatment [12]. The present situation is even more threatening when considering the stagnation in the development and approval of novel antimicrobial agents to treat these pathogens [13]. Therefore, an immediate and continual search for new antimicrobial agents effective against drug-resistant bacteria, preferentially with a lower risk of resistance formation, is urgently needed.

**Figure 1:**
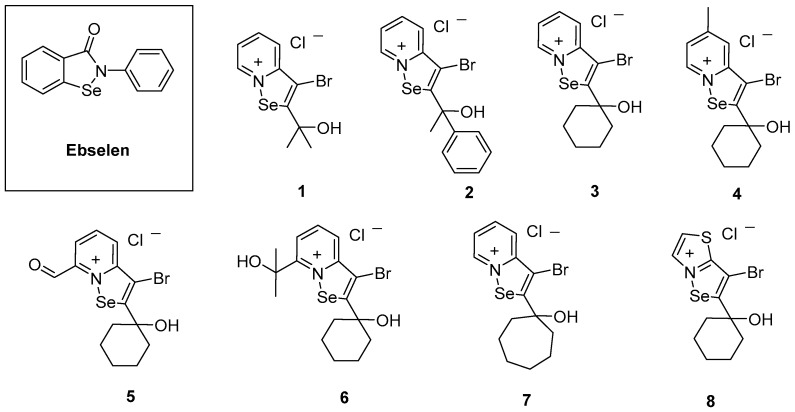
Chemical structures of ebselen (in the insert) and the selenazolinium salts **1**–**8** investigated.

In the last few years, selenium-based compounds have received significant attention due to their unique biological properties which could have multiple prospective applications in clinical practice [14]. The organoselenium compound ebselen (2-phenyl-1,2-benzisoselenazol-3(2*H*)-one, Figure 1), in particular, is a promising agent for the therapy of various health disorders [15,16] and is presently undergoing Phase III clinical trials in patients suffering from acute ischemic stroke and cortical infarct due to its pharmacological efficacy and favourable safety profile [17,18]. Ebselen, like many other organic selenium compounds, is redox-active and able to modify cysteine residues in proteins and enzymes effectively and selectively. In the presence of elevated levels of reactive oxygen species (ROS), it can also “turn catalytic”, and its pronounced glutathione peroxidase (GPx)-like activity promotes widespread interactions with cysteine residues of proteins belonging to the cellular thiolstat [19], hence resulting in significant decreases of intracellular protein thiols and activation of various redox-controlled cellular pathways [20]. In addition to various pro- and antioxidant actions associated traditionally with ebselen, recent studies have also discovered an excellent bactericidal action against the Gram-positive ESKAPE species *E. faecalis/E. faecium* and *S. aureus*. Curiously, ebselen lacks any major activity against Gram-negative ESKAPE pathogens [21]. This may not be extraordinarily surprising, as ebselen is not the most reactive amongst the selenium compounds frequently discussed today.

The overarching rationale of this study has therefore been the evaluation of ebselen-like selenium compounds with similar structural features and specificity towards nucleophilic attack by thiols, yet with improved reactivity. Within this context, one particular strategy to “improve” the reactivity of the Se–N bond towards cysteine thiols is the introduction of a positive charge which increases the electrophilic behaviour of the bond.

As part of this strategy, and during the search for new ebselen analogues containing fused rings with an endocyclic Se–N bond, a series of selenazolinium salts were obtained [22]. Eight of these compounds (**1**–**8**, Figure 1), have been investigated here for their antibacterial properties against a variety of *S. aureus* clinical isolates, as well as representative strains of clinically relevant Gram-negative ESKAPE bacteria. In addition, initial pharmaceutical safety studies have been carried out, and the most active compounds have been investigated regarding their potential mechanisms of action.

## 2. Results and Discussion

### 2.1. Chemical Synthesis

Synthesis of 3-bromo-selenazolopyridinium chlorides **1**–**7** and 3-bromo-2-(1-hydroxycyclohexyl)[1,2]selenazolo[2,3-*b*]thiazolinium chloride **8** (Figure 1) was described elsewhere [22].

### 2.2. In Vitro Antibacterial Activity

The in vitro antibacterial activities of the selenazolinium compounds (**1**–**8**) were evaluated for 25 strains of ESKAPE bacteria, including 11 Gram-positive strains (Table 1) and 14 Gram-negative microbes (Table 2) with a variety of clinical characteristics.

#### 2.2.1. Antibacterial Activity against *S. aureus* Strains

Antimicrobial activities of compounds **1**–**8** were investigated against 11 strains of *S. aureus* (Gram-positive bacteria) in comparison to ebselen and oxacillin. (Table 3). Results demonstrate that all selenocompounds (**1**–**8**) exhibited excellent antibacterial activities against all *S. aureus* strains employed in this study, with MIC values (0.31–3.44 µg/mL) in a similar range as that of ebselen (0.28–2.24 µg/mL). The same antibacterial effect of compounds **1**–**8** was observed against both the methicillin-susceptible *S. aureus* isolates ATCC 25923, MM-O058, and MM-N072 (MICs = 0.35–3.44 µg/mL) as well as the methicillin-resistant *S. aureus* isolates USA300 LAC (CA-MRSA), 5328 (LA-MRSA), LG-N017, MM-O021, R45 CC22, R45 CC45, HEMSA 5 (HA-MRSA), and Mu50 (VISA) (MIC = 0.31–3.44 µg/mL). Most of the compounds, except the aldehyde analogue (5), were more active against all MDR and XDR strains as compared to the reference strain (ATCC 25923, Table 3). This selectivity for the MDR and XDR (extensively drug-resistant) strains—paired with high activity at low concentrations—turns these compounds into a promising tool in the fight against drug-resistant bacteria [23]. Moreover, in all cases, compounds **1**–**8** exhibited significantly higher antimicrobial activity against MRSA clinical isolates when compared to the standard drug oxacillin. Among selenazolinium salts, compounds **1** and **6** represented the most effective antimicrobials with MIC values below 1 μg/mL against all but one MRSA strain, namely HEMSA 5.

#### 2.2.2. Antibacterial Activity against Gram-Negative Bacteria

Subsequently, selenocompounds **1**–**8** were evaluated for their possible inhibitory effects against four particularly problematic Gram-negative bacterial species belonging to ESKAPE pathogens (Table 2), and compared to ebselen (Table 4). At least three isolates of *K. pneumoniae*, *Acinetobacter* spp., *P. aeruginosa*, and *E. coli*, including 3 or 4 MRGN strains, were employed in the MIC experiments. Bacteria identified as 3 or 4 MRGN are resistant to three or four out of four classes of antibiotic groups used in the treatment of infections that they cause, respectively [24].

Compounds with MICs < 5 μg/mL were considered as highly active ones. In stark contrast to ebselen, all selenoazolinium salts (**1**–**8**) exhibited significant antibacterial activities against at least two Gram-negative ESKAPE strains. The highest sensibility for compounds **1**–**8** was observed for *Acinetobacter* spp., whereas the reference strain of *P. aeruginosa* (ATCC 27853) was sensitive only to compound **1**. Interestingly, compounds **1**–**8** were significantly more active in case of MDR *P. aeruginosa* strains when compared to the reference strain. Apart from the ATCC 27853 strain, all selenoazolinium compounds (**1**–**8**) exhibited MICs significantly lower than those of ebselen. Among the selenazolinium salts, compound **1** demonstrated excellent activities against all ESKAPE strains tested (MICs = 0.31–2.48 µg/mL). A high antibacterial potency was also observed for compounds **3**, **4**, and **7**, as these selenium compounds were active against 3 MRGN strains of *E. coli* and the 4 MRGN *K. pneumoniae* isolate (Table 4).

### 2.3. Results of Pharmaceutical Safety 

In order to assess some central drug-safety properties for the selenium agents (**1**–**8** and ebselen), and bearing in mind that certain inorganic-selenium compounds such as selenite (SeO_3_^2−^) interact unfavourably with DNA, all relevant compounds were investigated for their possible mutagenic properties in vitro employing the microtiter Ames test [25,26,27,28,29]. 

**Figure 2:**
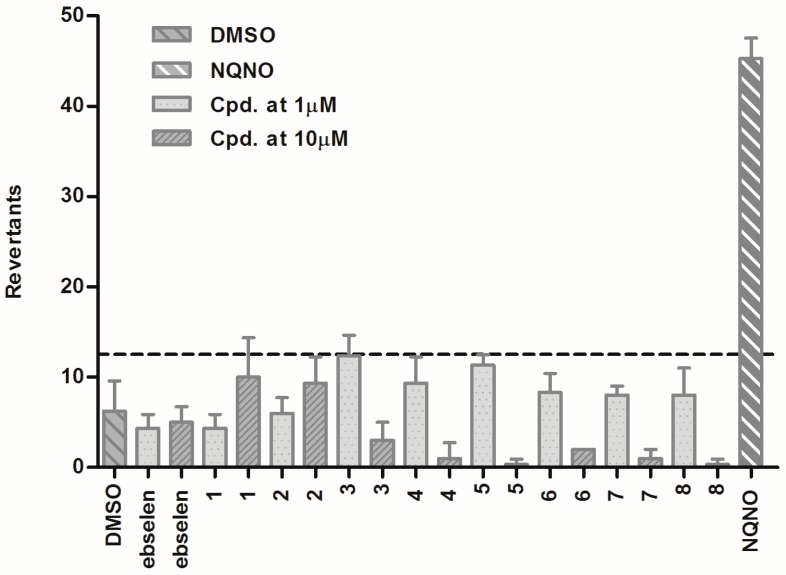
Results of the Ames liquid microtiter test and determination of the mutagenic potential; DMSO (1%)—negative control; ebselen-reference compound; NQNO (4-nitroquinoline-*N*-oxide)—mutagenic agent at concentration 0.5 µM; **1**–**8**—selenocompounds at concentrations 1 µM and 10 µM, respectively; —baseline defining the mutagenicity threshold (over the line).

Each experiment was performed in triplicate, and results were given in terms of mutagenic index (MI), which is the quotient of the number of revertant colonies induced in a test sample and the number of revertants in a negative control (media with 1% DMSO). A compound is considered mutagenic if MI is above 2.0 [30,31]. According to the results obtained (Figure 2), neither selenazolinium salts (**1**–**8**) nor ebselen appeared to cause any significant mutagenic changes at the concentrations used (1 µM and 10 µM), resulting in MI below 2.0, whereas the MI value for reference NQNO (0.5 μM) was 7.24 (Table 5). When comparing the MI values obtained between the two concentrations tested (1 µM and 10 µM), however, an evident decrease of MI values for the higher concentration of compounds **4**–**8** can be noted (Table 5). This decrease is connected with the cytotoxic activity of **4**–**8** against the *Salmonella typhimurium* TA100 strain, confirmed at 10 µM by Binominal B-values calculated according the manufacturer protocol, where B ≤ 0.01 indicates the occurrence of cytotoxic events (Figure 2, Table 5).

### 2.4. Studies on the Possible Mode of Antimicrobial Action

Whilst the results obtained so far point towards a considerable and widespread antibacterial activity associated with virtually all of the selenazolinium compounds under investigation, they do not reveal any information regarding the possible underlying mode(s) of action. Based on the chemical structures of these compounds, and their particular reactivity as thiol-selective electrophiles, one may expect a certain “redox link” that has been investigated in more detail. Such a link may constitute, for instance, in the production of ROS, a loss of thiols or a—possibly catalytic—oxidation of specific thiol groups in particularly redox-sensitive proteins and enzymes of the cellular thiolstat [32,33,34].

#### 2.4.1. Evaluation of ROS Formation

In order to analyse the effect of the selenazolinium salts (**1**–**8**) on intracellular oxidative stress production in *S. aureus*, the 2′,7′-dichlorofluorescein diacetate assay (DCFH-DA assay) was performed. This assay is used routinely in the context of human cell culture and can also be applied to bacteria. For this purpose, the impact of the most active compounds identified in the MICs assays (**1** and **6**) and ebselen on intracellular ROS concentrations was determined with the reference *S. aureus* strain ATCC 25923 and the clinical XDR-MRSA isolate HEMSA 5 (Figures S1–S3, Supplementary section). Rather unexpectedly, we observed that neither compound **1** nor compound **6** were able to increase ROS levels in the *S. aureus* isolates tested.

#### 2.4.2. Reactivity of the Compounds with Thiol Groups

**Figure 3:**
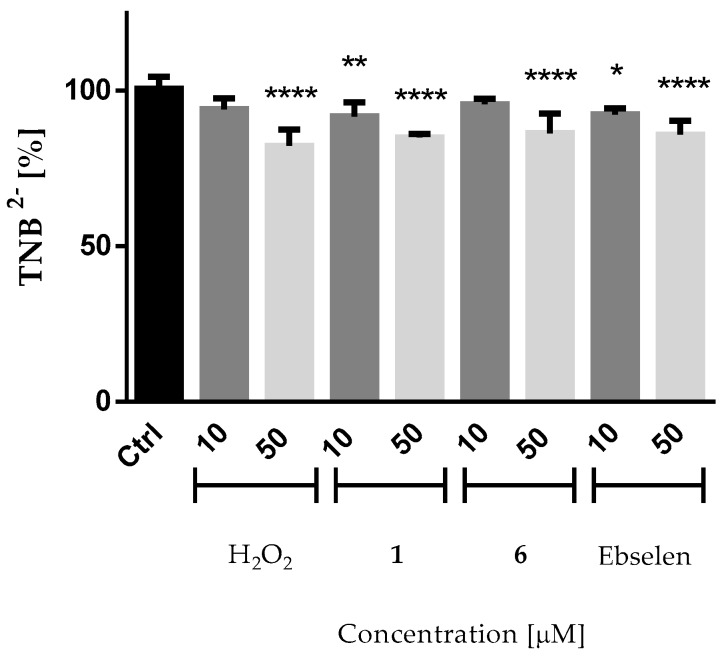
Estimation of levels of thiol residues of 100 µM *N*-acetyl-l-cysteine in the presence of different concentrations of compounds **1**, **6**, or ebselen by the DTNB assay. Hydrogen peroxide served as positive control in the study. The decrease in 2-nitro-5-thiobenzoate (TNB) indicates the ability of the compounds/H_2_O_2_ to modify free sulfhydryl groups of various amino acids of the proteins. It should be noted that compounds have been added in substoichiometric amounts compared to *N*-acetyl-l-cysteine to account for possible thiol oxidation cascades. Statistical significance was assessed by one-way ANOVA (mean ± SD, *n* = 5 replicates) followed by Dunnett’s multiple comparisons test. * *p* ≤ 0.05, ** *p* ≤ 0.01 and **** *p* ≤ 0.0001 compared with the control.

Recent studies have shown that many chalcogen compounds do not generate ROS per se but instead interfere with their removal by consuming thiol groups [35,36,37]. Indeed, selenium-based molecules have been reported to spontaneously react with various biological thiols, including GSH and cysteine-containing proteins. We therefore performed a standard Ellman’s Reagent DTNB assay in order to explore the consumption of thiols as possible mode of antibacterial activity of the compounds tested (Figure 3).

The ability of the selenium-based compounds to consume thiol groups was determined with *N*-acetyl-l-cysteine, which was exposed to the compounds and whose remaining thiol groups were subsequently quantified with Ellman’s Reagent DTNB. A loss in thiol groups due to the presence of the selenium compounds would be noted by less TNB production assuming that the products of the reaction of *N*-acetyl-l-cysteine with the selenium compounds do not react with DTNB. As shown in Figure 3, the addition of the compounds (**1** and **6**) at concentrations as low as ≤50 µM to the *N*-acetyl-l-cysteine solution significantly decreased the levels of TNB, indicating that the compounds are able to modify cysteine, and hence may also be able to attack various proteins and enzymes of the cellular thiolstat of the bacteria. The cysteine-modifying activities of the compounds were comparable to or even higher than one of the reference molecules—hydrogen peroxide. Interestingly, the more pronounced effect was observed for compound **1**—which was also very active against bacteria—followed by ebselen and, to a lesser extent, by compound **6**.

### 2.5. Structure–Activity Relationship Discussion

Overall, the studies performed with selenazolinium salts **1**–**8** have identified this rather unusual class of selenium compounds as very reactive chemically and very active biologically. It is highly probable that the extraordinary electrophilic behaviour associated with the positively charged Se–N motif is responsible for the excellent growth inhibitory activities on all MDR *S. aureus* strains. Indeed, the selenazolinium salts **1**–**8** showed a comparable influence on *S. aureus*, often exceeding the one of ebselen, and in all MRSA strains were considerably more active when compared to the standard antibiotic oxacillin. Two compounds, the 3-bromo-2-(1-hydroxy-1-methylethyl)[1,2]selenazolo[2,3-*a*]pyridinium chloride (**1**) and the 3-bromo-2-(1-hydroxycyclohexyl)-7-(2-hydroxypropan-2-yl)[1,2]selenazolo[2,3-*a*]pyridinium chloride (**6**) displayed an even stronger antimicrobial effect than ebselen against two of the MRSA strains tested (LG-N017 and R45 CC22). It is worth emphasizing that their antistaphylococcal activities in form of MICs are superior to those recently reported for fluoroquinolones and their thiolated analogues [5]. Indeed, the activity of the selenazolinium salts is comparable to that of the most active agents produced by the latest lines of investigation, e.g., 9,13-disubstituted berberine derivatives [38] or polyhalogenated 2-phenylbenzimidazoles [39].

The studies on the possible mechanisms of action against MRSA strains identified a similar behaviour of the selenazolinium compounds (**1** and **6**) and ebselen in all three assays indicating that the mode of action of the compounds is related to an extensive modification of thiol groups, possibly in cysteine-containing cellular proteins that are crucial for bacterial survival and growth. Interestingly, in both cases, i.e., the 3-bromo-selenoazolinium compounds and ebselen, there was only a slight strain-related discrimination in the antibacterial effects observed, and an even more potent efficacy in the case of MDR bacteria than in the corresponding reference strains. In contrast to oxacillin, it is possible that such Se–N endocyclic selenocompounds can overcome certain MDR mechanisms. There may be various reasons for such behaviour. One may, for instance, consider a widespread and simultaneous attack of such reactive selenium compounds on various redox-sensitive cysteine proteins, hence avoiding the kind of resistance associated with “single target” drugs. Alternatively, a significant consumption of cellular thiols may also trigger unfavourable intracellular signalling processes which may eventually harm the bacterium affected. In any case, the considerably high activity, especially against MDR-strains, is of great interest for the current search of antimicrobial agents.

Here, an even more important finding of these studies is the high potency of the compounds **1**–**8** against Gram-negative ESKAPE bacteria, which clearly distinguishes these selenazolinium compounds from ebselen and places them on par with the best anti-ESKAPE agents found recently, such as isothiazolone [40] or bis-cyclic guanidine compounds [41]. The results obtained here with 14 different Gram-negative strains evidently confirm the promising properties of the entire group of both, the 3-bromo-selenazolopyridinium chlorides (**1**–**7**) and 3-bromo-2-(1-hydroxycyclohexyl)[1,2]selenazolo[2,3-*b*]thiazolinium chloride (**8**), against both types of ESKAPE strains, with special accent on the selenazolopyridinium compounds **1** and **3** (Figure 1, Table 2 and Table 3).

Although it is difficult to discuss probable mechanisms of action, in this case, it is rather obvious that the charged endocyclic Se–N bond is beneficial for the action against Gram-negative pathogens. In contrast, ebselen is not active against the latter group of bacteria. Nonetheless, not all selenazolinium salts were equally active and some differences in activity have been observed within the group (**1**–**8**). Thus, decreased activities were observed for the hydroxycyclohexyl derivatives substituted at position 7 with formyl (**5**) or hydroxyl-alkyl (**6**) moieties. A very simple structure–activity relationship (SAR) analysis suggests that substituents at the pyridine part of the fused rings of compounds **1**–**7** may influence the action on Gram-negative pathogens. Thus, a substitution at position 7 could be responsible for a decrease of antibacterial action. The most active compounds **1** and **3** are not substituted within the pyridine part of the fused rings as well as they include 1-hydroxy-1-methylethyl- or 1-hydroxycyclohexyl terminal fragments, respectively (Figure 1, Table 2 and Table 3). It is not entirely clear yet, however, which of the structural properties are really responsible for the outstanding action observed for some of the compounds, since both the unsubstituted pyridine fragment and the 1-hydroxycyclohexyl one are also present in the less active compounds (**2**, **4**–**6** and **8**, respectively). In the case of activity against *S. aureus*, the most active compounds (**1** and **6**) are also the more hydrophilic. The first one (**1**) possesses the smallest acyclic alcohol moiety, whereas compound **6** includes two hydroxyl groups.

The excellent results obtained for compounds **1**–**8** in the assays against a panel of 25 ESKAPE bacterial strains demands further studies for this group of unique compounds in order to evaluate the pharmaceutical safety profile. In this context, we have already applied the Ames mutagenicity assays, a gold standard in the initial steps of drug R&D process. The results of low mutagenicity risk for compounds **1**–**8** obtained, in resemblance to ebselen, indicate that the new selenazolinium compounds can be considered as potential candidates for a new drug that is successful in the battle against the most problematic multidrug-resistant pathogens. Still, a high electrophilic reactivity may also imply a more random reactivity, for instance, also against human cells, and such issues related to selectivity should be considered in future studies.

## 3. Materials and Methods

### 3.1. Microbiological Assays

#### 3.1.1. Chemical Compounds

The selenazolinium salts: 3-bromo-2-(1-hydroxy-1-methylethyl)[1,2]selenazolo[2,3-*a*]pyridinium chloride (**1**), 3-bromo-2-(1-hydroxy-1-phenylethyl)[1,2]selenazolo[2,3-*a*]pyridinium chloride (**2**), 3-bromo-2-(1-hydroxycyclohexyl)[1,2]selenazolo[2,3-*a*]pyridinium chloride (**3**), 3-bromo-2-(1-hydroxycyclohexyl)-5-methyl[1,2]selenazolo[2,3-*a*]pyridinium chloride (**4**), 3-bromo-7-formyl-2-(1-hydroxycyclohexyl)[1,2]selenazolo[2,3-*a*]pyridinium chloride (**5**), 3-bromo-2-(1-hydroxycyclohexyl)-7-(2-hydroxypropan-2-yl)[1,2]selenazolo[2,3-*a*]pyridinium chloride (**6**), 3-bromo-2-(1-hydroxycycloheptyl)[1,2]selenazolo[2,3-*a*]pyridinium chloride (**7**), and 3-bromo-2-(1-hydroxycyclohexyl)[1,2]selenazolo[2,3-*b*]thiazolinium chloride (**8**) were synthesized according to the procedures described previously [22].

Ebselen was purchased from Sigma-Aldrich (St. Louis, MO, USA). The stock solutions of the compounds tested were prepared in DMSO/H_2_O and stored at −20 °C until used. Furthermore, the following solvents and chemical compounds were employed in our studies: 2′,7′-dichlorofluorescein diacetate (DCFH-DA) and oxacillin (Sigma-Aldrich, St. Louis, MO, USA); 2-azobis(2-amidinopropane) dihydrochloride (AAPH; Sigma-Aldrich, Steinheim, Germany); H_2_O_2_ (Life Technologies, Eugene, OR, USA); 5,5′-dithiobis-2-nitrobenzoic acid (DTNB, Ellman reagent; Sigma-Aldrich, St. Louis, MO, USA); *N*-acetyl-l-cysteine (Alfa-Aesar, Karlsruhe, Germany); ampicillin (Polfa Tarchomin S.A., Warszawa, Poland); 4-nitroquinoline-*N*-oxide (NQNO; Sigma-Aldrich, Munich, Germany); DMSO and bromocresol purple (Sigma-Aldrich, Munich, Germany); Beef extract, l-histidine monochloride, and d-biotin (Bioshop, 5480 Mainway, Burlington, Ontario, Canada); peptone from casein (Merck, Darmstadt, Germany); KH_2_PO_4_, K_3_PO_4_, (NH_4_)_2_SO_4_, MgSO_4_ × 7H_2_O, NaCl, trisodium citrate dehydrate, and d-glucose (Chempur, Piekary Śląskie, Poland).

#### 3.1.2. Bacterial Strains

Twenty-five bacterial strains used in this study are listed in Table 1. *S. aureus* ATCC^®^25923^TM^ and MRSA HEMSA 5 were obtained from the Institute of Hygiene and Tropical Medicine, Universidade Nova de Lisboa, Lisbon, Portugal; and the *A. baumannii* isolate AB 4184/2/5 was obtained from Department of Pharmaceutical Microbiology, Faculty of Pharmacy, Jagiellonian University Medical College, Cracow, Poland. The remaining bacterial strains were obtained from the stock collection of the Institute of Medical Microbiology and Hygiene, Saarland University, Homburg, Germany. *S. typhimurium* TA100 strain (Xenometrix, Allschwil, Switzerland) with the base pair substitution (*hisG46* mutation, which target is GGG) was used for the Ames assays indicative of mutagenicity.

#### 3.1.3. Antimicrobial Susceptibility Testing

The minimal inhibitory concentration tests were performed by the standard microdilution method in cation-adjusted Mueller Hinton II Broth (MHB II, Becton-Dickinson, Heidelberg, Germany) according to Clinical and Laboratory Standards Institute (CLSI) recommendations [42]. Antibacterial activities against *S. aureus* strains were evaluated in comparison with the β-lactam antibiotic oxacillin. The MICs of compounds **1**–**8** and ebselen were recorded after 20 h incubation at 37 °C. The antibacterial effect was determined in triplicate in at least three independent experiments.

#### 3.1.4. Determination of Intracellular Oxidative Stress Levels via the DCFH-DA Assay

Reactive oxygen species (ROS) production in *S. aureus* strains ATCC 25923 and MRSA HEMSA 5 under exposure to a given selenium compound was measured by using the redox-sensitive fluorescent indicator dye DCFH-DA, according to the protocol published previously [43]. The concentrations of the compounds were used at levels ranging from 1/2 to 2× their MICs. AAPH at the final concentration of 50 mM was included as positive control in the assay. The fluorescence intensity was detected at 5 min intervals over a 60 min period using a microplate reader (EnSpire, PerkinElmer, Waltham, MA, USA), with an excitation wavelength of *λ*_ex_ = 480 nm and an emission wavelength of *λ*_em_ = 525 nm.

#### 3.1.5. DTNB Assay

The assay was carried out following the procedure described earlier with a few modifications [44,45]. DTNB was used to quantify the concentration of free thiol groups in the sample. The method is based on the reaction of this aromatic disulfide with aliphatic thiol groups of a compound to form a mixed disulfide and 2-nitro-5-thiobenzoate (TNB), which ionizes to the TNB^2−^ dianion at neutral or alkaline pH. The reaction is rapid and stoichiometric, the addition of 1 mol of thiol-containing compound leads to the release of 1 mol of TNB. The latter gives an intense yellow color that can be quantified spectrophotometrically at the wavelength of 412 nm. The DTNB assay was initiated by mixing 180 µL of 100 µM *N*-acetyl-l-cysteine in 0.1 M phosphate buffer solution with either 10 µL of 200 µM, or 1 mM or 2 mM solution of ebselen or selenazolinium salts exhibiting the highest antibacterial activity in the previous studies (compound **1** and **6**), or hydrogen peroxide, which was used as a positive control in the assay. After 40 min incubation, 10 µL of DTNB (4 mM) solution was added and the decrease in absorbance was measured spectrophotometrically by using a microplate reader (EnSpire, PerkinElmer, Waltham, MA, USA) at a wavelength of 412 nm for 10 min at 25 °C. One should note that compared to *N*-acetyl-l-cysteine, the compounds were added in sub-stoichiometric amounts, as it is possible that such selenium agents react with more than one equivalent of thiols. It is therefore not expected that all thiols of *N*-acetyl-l-cysteine are eventually consumed in this assay.

#### 3.1.6. Ames Test

The alternative Ames test adapted to the HTS microplate format (Xenometrix AG, Allschwil, Switzerland) was performed using the histidine-dependent *Salmonella typhimurium* strain TA100, according to the previously described method of Kamber et al. [25,26,27,28,29]. All the compounds tested and the reference (ebselen) were evaluated at final concentrations of 1 µM and 10 µM (in the well). NQNO was applied as a positive standard mutagen control (0.1 µM, 0.5 µM) [29]. Finally, the results were counted manually and by the use of microplate reader. The mutagenic index (MI) was calculated next, as the quotient of the number of revertant colonies induced in a test sample and the number of revertants in a negative control (media with 1% DMSO). A compound is considered mutagenic if its MI is above 2.0. The Binomial B-values were calculated according to the protocol provided by Xenometrix AG.

## 4. Conclusions

In the present study, a novel group of selenazolinium salts displaying excellent in vitro activity against ESKAPE pathogens has been described. Similar to ebselen, these reactive selenium species (RSeS) have demonstrated great potential against 11 strains of *S. aureus*, including multidrug-resistant MRSA and VISA clinical isolates. Yet in stark contrast to ebselen, the selenazolinium compounds also displayed a significant antibacterial action against various members of the Gram-negative ESKAPE family, including *K. pneumoniae*, *A. baumannii*, *P. aeruginosa*, and *E. coli*. Moreover, a beneficial pharmaceutical safety for all selenoazolinium compounds (**1**–**8**) has been confirmed in the Ames mutagenicity assays. These differences between ebselen on one side and the selenazolinium salts on the other may be explained by differences in reactivity and cellular target(s). Indeed, our preliminary mechanistic studies indicated that the mode of action for the Se-compounds is likely to be associated with an alteration of intracellular levels of glutathione and cysteine thiols of different proteins in the bacterial cells, hence supporting the idea that such compounds interact with the intracellular thiolstat. Future studies may reveal if this reaction is stoichiometric, or—as anticipated from the low amounts employed—catalytic with respect to pre-existing ROS and thiols. It is also possible that the selenazolinium salts react more than once, and that the active species is not the salt itself but possibly a reactive seleno-sulfide intermediate formed as part of the sequestration by and modification with GSH. Such investigations may also differentiate between ebselen, which is generally seen as an “antioxidant” promoting cell survival by reducing ROS in the presence of GSH, and the kind of oxidizing RSeS employed here, which, due to their high(er) reactivity, seem to attack thiol residues more randomly, hence modulating the level of GSH yet also affecting proteins and enzymes.

Taking into account such promising properties, we can conclude that the compounds with the selenazolinium scaffold represent a very promising chemical family in the ongoing search for new drug candidates that efficiently combat infections caused by highly resistant ESKAPE bacteria. Thus, the series is worth passing on for further stages of the drug research and development processes and also for more detailed investigations of the underlying mode(s) of action. At the same time, the Se–N motif may be refined and “tuned” further to enhance activity and selectivity.

## Figures and Tables

**Table 1:** Characteristics of the ESKAPE Gram-positive strains used in this study.

Bacterial Strain	Relevant Phenotype *
*Staphylococcus aureus*	
ATCC 25923	Reference strain, CC5, MSSA
MM-O058	Clinical isolate, CC121, MSSA, MDR
MM-N072	Clinical isolate, CC152, MSSA, MDR
HEMSA 5	Clinical isolate, MRSA, XDR
LG-N017	Clinical isolate, CC5, MRSA, MDR
MM-O021	Clinical isolate, CC8, MRSA, MDR
R45-CC45	Clinical isolate, CC45, MRSA
R46-CC22	Clinical isolate, CC22, MRSA
USA300 LAC	Clinical isolate, CC8, CA-MRSA, MDR
5328	Clinical isolate, CC398, LA-MRSA
Mu50	Clinical isolate, CC5, MRSA, VISA

* CC, clonal complex; MSSA, methicillin-susceptible *S. aureus*; MRSA, methicillin-resistant *S. aureus*; CA-MRSA, community-acquired MRSA; LA-MRSA, livestock-associated MRSA; VISA, vancomycin-intermediate *S. aureus;* MDR, multidrug-resistant; XDR, extensively drug-resistant [23].

**Table 2:** Characteristics of the ESKAPE Gram-negative strains used in this study.

Bacterial Strain	Relevant Phenotype
*Klebsiella pneumoniae*	
NRZ-00103	Reference strain, KPC-2, MDR, 4 MRGN
KP 21513017	Clinical isolate
KP 1963584	Clinical isolate, OXA-2, MDR, 4 MRGN
*Acinetobacter* spp.	
AC 2151300	Clinical isolate, *A. ursingii*
AC 1995594	Clinical isolate, *A. baumannii complex*
AB 4184/2/5	Clinical isolate, *A. baumannii*
*Pseudomonas aeruginosa*	
ATCC 27853	Reference strain
PA T18	Clinical isolate, MDR, 3 MRGN
PA 54	Clinical isolate, MDR
PA 58	Clinical isolate, MDR
*Escherichia coli*	
NCTC 13351	Reference strain, TEM-3
EC 2151612	Clinical isolate, MDR, 3 MRGN
EC 1995591	Clinical isolate, MDR, 3 MRGN
EC 1227107	Clinical isolate, MDR, 3 MRGN

3/4 MRGN bacteria, multidrug-resistant Gram-negative bacteria. The description 3 or 4 MRGN refers to the classification of multidrug-resistant Gram-negative bacteria created by the Commission for Hospital Hygiene and Infectious Disease Prevention (KRINKO) of the Robert Koch-Institute (RKI) in order to outline the resistance pattern of these pathogens. Bacteria identified as 3 or 4 MRGN are resistant to three or four out of four classes of antibiotic groups used in the treatment of infections that they cause, respectively [24]; KPC-2, carbapenemase KPC-2; OXA-2, β-lactamase OXA-2; TEM-3, extended-spectrum β-lactamase TEM-3.

**Table 3:** Antibacterial activities (MICs) of compounds **1**–**8** against different strains of *S. aureus*.

*S. aureus* Strain	MIC (µg/mL)									
	OXA	1	2	3	4	5	6	7	8	Ebselen
ATCC 25923	0.25	1.24	1.72–3.44	1.4	1.45	0.72–1.44	0.36–0.72	1.44–2.88	2.88	0.56
MM-O058 *	0.25	0.62	0.86	0.35–0.7	0.36–0.73	0.72	0.36–0.72	0.36–0.72	0.36–0.72	0.56–1.12
MM-N072 *	0.25	0.62–1.24	3.44	0.7–1.4	0.73–1.46	0.72	0.36–0.72	0.72	1.44–2.88	0.56–1.12
USA300 LAC *	12	0.62	3.44	0.7	0.73	0.72–1.44	0.72–1.44	0.36–0.72	0.72–1.44	0.56–1.12
5328	4	0.62–1.24	3.44	1.4	1.45	1.44–2.88	0.72–1.44	0.72–1.44	1.44–2.88	1.12–2.24
LG-N017 *	12	0.62–1.24	0.86–1.72	0.35–0.7	0.73–1.45	0.72–1.44	0.36–0.72	0.36–1.44	0.72–1.44	1.12–2.24
MM-O021 *	64	0.31–0.62	1.72–3.44	0.35–0.7	0.36–0.73	0.36–0.72	0.36–0.72	0.36–0.72	0.72–1.44	0.56–1.12
R45 CC22	64	0.31	0.86–1.72	0.35	0.36–0.73	0.36–0.72	0.36	0.36–0.72	0.36–0.72	1.12
R45 CC45	4	0.31–0.62	1.72–3.44	0.7–1.4	0.36–0.73	0.72	0.36–0.72	0.72	1.44	0.56
HEMSA 5 **	128	1.24	1.72	1.4	1.45	1.44	0.72	0.72–1.44	2.88	2.8
Mu50 ***	256	0.31–0.62	0.43–0.86	0.7–1.4	0.36–0.73	0.36–0.72	0.36	0.36–0.72	0.72–1.44	0.28

* MDR, multidrug-resistant isolates; ** XDR, extensively drug-resistant isolate; *** VISA strain. Oxacillin (OXA) was used as reference β-lactam antibiotic.

**Table 4:** MIC values of the compounds **1**–**8** and ebselen against Gram-negative ESKAPE strains.

	Bacterial Strain	MIC (µg/mL)								
		1	2	3	4	5	6	7	8	Ebselen
*K. pneumoniae*	1	1.24 *	14	1.4–2.8	2.88–5.76	46	46	2.88	5.76	≥143
	2	0.62–1.24	6.88–14	1.4	2.88	35	17	2.88	5.76	108
	3	0.62	6.88	1.4	2.88	17	17	1.44–2.88	2.88	72
*Acinetobacter* spp.	4	0.31–0.62	0.86	0.35–0.7	0.36–0.72	2.88	1.44	0.36–0.72	1.44	18
	5	0.62	1.72–3.44	1.4	0.72	2.88–5.76	2.88	0.72	1.44–2.88	27
	6	0.31	0.86	0.35	0.36	2.88	0.72–1.44	0.36	1.44	18
*P. aeruginosa*	7	2.48	110	5.60–11	5.76–12	69	138	17	23	72
	8	0.31	1.72	0.7	1.44	12	12	0.72	1.44–2.88	18
	9	0.62–1.24	21	1.4–2.8	5.76	12	69	5.76	5.76–12	108
	10	0.62	6.88–14	1.4	2.88	17	23	2.88	5.76	27
*E. coli*	11	1.24–2.48	6.88–14	1.4	2.88	17	12	2.88	5.76	108
	12	1.24–2.48	6.88	1.4	2.88	17	12	1.44–2.88	5.76	72
	13	2.48	6.88–14	1.44–2.8	2.88	23	12	2.88	5.76	72
	14	1.24–2.48	14	2.8	5.76	23	17	2.88	5.76–12	54

* Particularly potent antibacterial activities (MIC < 5 μg/mL) are underlined. In case of MIC ≥ 10 µg/mL, the MIC values are expressed rounded to integers (see Supplementary for details). Bacterial strains—*K. pneumoniae*: (1) NRZ-00103, (2) KP 21513017, (3) KP 1963584; *Acinetobacter* spp.: (4) AC 2151300, (5) AB 1995594, (6) AB 4184/2/5; *P. aeruginosa*: (7) ATCC 27853, (8) PA T18, (9) PA54, (10) PA58; *E. coli*: (11) NCTC 13351, (12) EC 2151612, (13) EC 1995591, (14) EC 1227107.

**Table 5:** Mutagenic index (MI) values for ebselen and tested compounds (**1**–**8**).

Cpd.	MI (1 µM)	B	MI (10 µM)	B
**Ebselen**	0.69	0.26	0.80	0.46
**1**	0.69	0.35	1.60	1.00
**2**	0.96	0.83	1.49	1.00
**3**	1.97	0.99	0.48	0.06
**4**	1.49	0.71	0.16	0.00
**5**	1.81	0.96	0.05	0.00
**6**	1.33	0.97	0.32	0.00
**7**	1.28	0.94	0.16	0.00
**8**	1.28	0.98	0.05	0.00

MI—mutagenic index values for ebselen and selenocompounds **1**–**8**, B—Binomial B-value.

## Supplementary Materials

The following are available online. Table S1: Details of MIC values of the compounds **1**–**8** and ebselen against Gram-negative bacteria, Figure S1: Generation of intracellular ROS in the reference *S. aureus* ATCC 25923 strain and in the clinical MRSA HEMSA 5 isolate upon exposure to the different concentrations of the compound 1, Figure S2: Generation of intracellular ROS in the reference *S. aureus* ATCC 25923 strain and in the clinical MRSA HEMSA 5 isolate upon exposure to the different concentrations of compound 6, Figure S3: Generation of intracellular ROS in the reference *S. aureus* ATCC 25923 strain and in the clinical MRSA HEMSA 5 isolate upon exposure to the different concentrations of ebselen.
