# Supplementary material for: Selenazolinium Salts as “Small Molecule Catalysts” with High Potency against ESKAPE Bacterial Pathogens
Source: Molecules. 2017 Dec 8;22(12):2174. doi: 10.3390/molecules22122174 (PMC6149925; doi:10.3390/molecules22122174)
Supplement: Supplementary file 1 [file molecules-22-02174-s001.pdf]

Supplementary

Table S1. Details of MIC values of the compounds **1-8** and ebselen against Gram-negative bacteria.

	Bacteria Strains	MIC* of the tested compounds (**1-8**) [μg/ml]								
		**1**	**2**	**3**	**4**	**5**	**6**	**7**	**8**	**Ebselen**
K. pneumoniae	**NRZ-00103**	1.24[1]	13.76	.4-2.8	2.88-5.76	46.08	46.08	2.88	5.76	≥ 143.35
	**KP 2151307**	0.62-1.24	6.88-13.76	1.4	2.88	23.04-46.08	11.52-23.04	2.88	5.76	71.68-143.36
	**KP 1963584**	0.62	6.88	1.4	2.88	11.52-23.04	11.52-23.04	1.44-2.88	2.88	71.68
Acinetobacter	**AC 2151300**	0.31-0.62	0.86	0.35-0.7	0.36-0.72	2.88	1.44	0.36-0.72	1.44	17.92
	**AB 1995594**	0.62	1.72-3.44	1.4	0.72	2.88-5.76	2.88	0.72	1.44-2.88	17.92-35.84
	**AB 4184/2/5**	0.31	0.86	0.35	0.36	2.88	0.72-1.44	0.36	1.44	17.92
P. aeruginosa	**ATCC 27853**	2.48	110.08	5.60-11	5.76-11.52	46.08-92.16	92.16-184.32	11.52-23.04	23.04	71.68
	**PA T18**	0.31	1.72	0.7	1.44	11.52	11.52	0.72	1.44-2.88	17.92
	**PA54**	0.62-1.24	13.76-27.52	1.4-2.8	5.76	11.52	46.08-92.16	5.76	5.76-11.52	71.68-143.36
	**PA58**	0.62	6.88-13.76	1.4	2.88	11.52-23.04	23.04	2.88	5.76	17.92-35.84
E. coli	**NCTC 13351**	1.24-2.48	6.88-13.76	1.4	2.88	11.52-23.04	11.52	2.88	5.76	71.68-143.36
	**EC 2151612**	1.24-2.48	6.88	1.4	2.88	11.52-23.04	11.52	1.4-2.88	5.76	71.68
	**EC 1995591**	2.48	6.88-13.76	1.4-2.8	2.88	23.04	11.52	2.88	5.76	71.68
	**EC 1227107**	1.24-2.48	13.76	2.8	5.76	23.04	11.52-23.04	2.88	5.76-11.52	35.84-71.68

* Particularly potent antibacterial activities (MIC < 5 μg/ml) are underlined.

3.3. Evaluation of ROS formation

To analyze the effect of the selenazolinium salts tested on intracellular oxidative stress production in *S. aureus* DCHFA assay was performed. For this purpose, the impact of the most active compounds which were identified in the previous studies (the compounds **1** and **6**) and ebselen on ROS release was determined in the reference *S. aureus* ATCC 25923 strain and the clinical isolate MRSA HEMSA 5 (**Figure S1-S3**)

a)

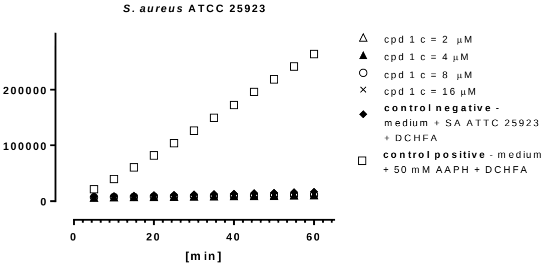

b)

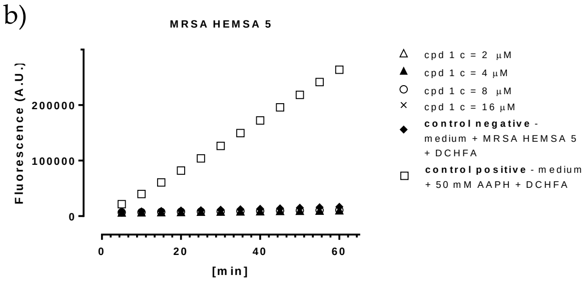

Figure S1. Generation of intracellular ROS in the reference *S. aureus* ATCC 25923 strain (a) and the clinical MRSA HEMSA 5 isolate (b) upon exposure to the different concentrations of the compound **1**. 2, 2′-azobis (2-amidinopropane) dihydrochloride (AAPH) was included as a positive control in the assay. The level of oxidative stress was detected by the use of fluorogenic dye 2', 7'-dichlorodihydrofluorescein diacetate (DCHFA) which in the presence of cellular esterases and ROS is converted to highly fluorescent 2', 7'-dichlorofluorescein (DCFA). Values represent means with standard deviation (SD) bars from at least four repeats. Statistical significances were calculated using a one-way ANOVA followed by Bonferroni's multiple comparison test (**Figure S1 a**, **b**: $p > 0.05$).

a)

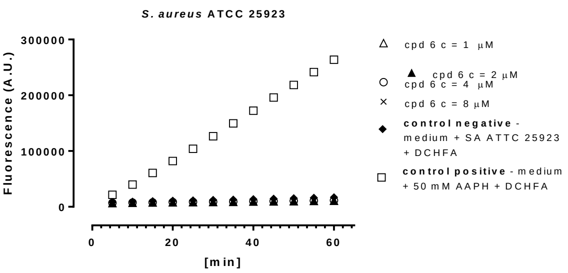

b)

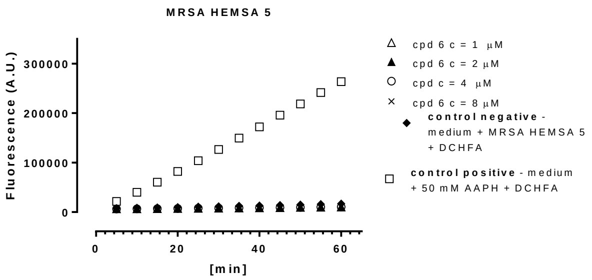

Figure S2. Generation of intracellular ROS in the reference *S. aureus* ATCC 25923 strain (a) and the clinical MRSA HEMSA 5 isolate (b) upon exposure to the different concentrations of compound **6**. For further details refer to the **Figure S1**.

a)

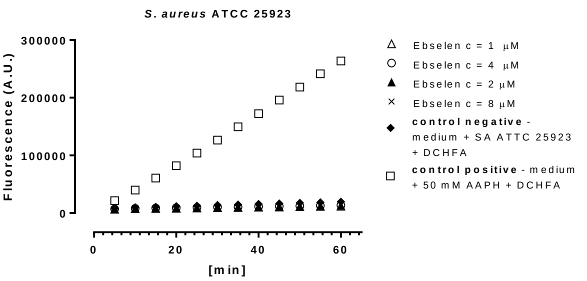

b)

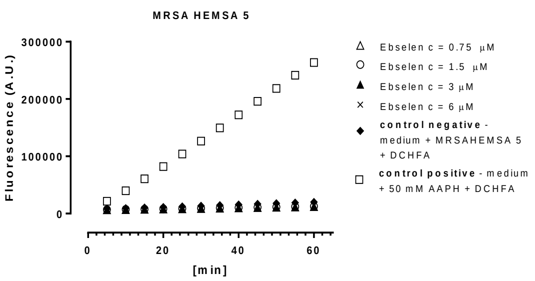

Figure S3. Generation of intracellular ROS in the reference *S. aureus* ATCC 25923 strain (a) and the clinical MRSA HEMSA 5 isolate (b) upon exposure to the different concentrations of ebselen. For further details, refer to the **Figure S1**.
